# Comprehensive characterization of clonality of driver genes revealing their clinical relevance in colorectal cancer

**DOI:** 10.1186/s12967-022-03529-x

**Published:** 2022-08-12

**Authors:** Jian Shi, Li Wang, Xiangzhe Yin, Lixia Wang, Lin Bo, Kailai Liu, Ke Feng, Shihua Lin, Yanjun Xu, Shangwei Ning, Hongying Zhao

**Affiliations:** 1 College of Bioinformatics Science and Technology, Harbin Medical University, Harbin, 150081 China; 2 Precision Medicine Institute, The First Affiliated Hospital, Sun Yat-Sen University, Guangzhou, China

**Keywords:** Subclonal mutation, Clonal mutation, Prognostic biomarkers, Driver genes, Immune evasion, Colorectal cancer

## Abstract

**Background:**

Genomic studies of colorectal cancer have revealed the complex genomic heterogeneity of the tumor. The acquisition and selection of genomic alterations may be critical to understanding the initiation and progression of this disease.

**Methods:**

In this study, we have systematically characterized the clonal architecture of 97 driver genes in 536 colorectal cancer patients from TCGA.

**Results:**

A high proportion of clonal mutations in 93 driver genes were observed. 40 genes showed significant associations between their clonality and multiple clinicopathologic factors. Kaplan–Meier analysis suggested that the mutation clonality of *ANK1*, *CASP8*, *SMAD2,* and *ARID1A* had a significant impact on the CRC patients' outcomes. Multivariable analysis revealed that subclonal *ANK1* mutations, clonal *CASP8* mutations, and clonal *SMAD2* mutations independently predicted for shorter overall survival after adjusting for clinicopathological factors. The poor outcome of the subclonal *ANK1* mutation may be caused by upregulation of IL4I1, IDO1, IFNG and MAPK12 which showed potential roles in tumor immune evasion through accumulation of immunosuppressive cells such as regulatory T cells and myeloid derived suppressor cells.

**Conclusion:**

These results suggested that the clonality of driver genes could act as prognostic markers and potential therapeutic targets in human colorectal cancer.

**Supplementary Information:**

The online version contains supplementary material available at 10.1186/s12967-022-03529-x.

## Introduction

Colorectal cancer (CRC) is the third most commonly diagnosed cancer in males and the second in females and the incidence of colorectal cancer is increasing in certain countries [1]. The molecular pathogenesis of colorectal cancer is characterized by the accumulation of genomic alterations in driver genes. Leveraging the next-generation sequencing technology, a large number of studies reveal that many genomic alterations locate in driver genes of colorectal cancers and influence the risk, onset, and progression of CRC. For example, the classical tumor progression model of sporadic CRC finds that frequent mutations occur in *APC*, *TP53*, *KRAS* gene, and *APC* mutation contributes to adenoma formation followed by the acquisition of *KRAS* mutations promoting the transition from intermediate adenomas to carcinomas with *TP53* inactivation as a late event [2]. Therefore, deciphering genomic aberrations information hide deep in the genome is key to unlock the puzzle of colorectal progression. Clonal heterogeneity, defined as the genomic diversity that exists within single tumors with clonal mutations presenting in all tumor cells and subclonal mutations presenting in a subset of tumor cells, is one of the most challenging issues in the field of current cancer research. Understanding clonal heterogeneity of driver genes is essential for developing personalized treatment for patients with colorectal cancers. Clonal heterogeneity resulting from the continuous accumulation of mutations during tumor progression reflects the relative time of mutation occurrence during tumorigenesis and can aid in predicting response and resistance to drugs [3, 4], influencing the clinical outcome of patients. However, These results remain to be further studied, because emerging evidence shows that tumors with the complex mutational composition are composed of heterogeneous tumor cells with clonal or subclonal mutations, which influences the progress of cancer evolution and impairs the prognosis of the patients [5–7]. Moreover, the presence of subclonal driver mutations influences a more aggressive evolution of the disease [8]. Taken together, these studies alert that clonal architecture may provide new insights into the prognostic influence of driver genes in CRC.

In the present study, we comprehensively characterized the clonal architecture of 97 driver genes in CRC and investigated their clinical impact according to somatic mutation and copy number alteration (SCNA) data of 536 CRC primary samples from The Cancer Genome Atlas (TCGA). Furthermore, we comprehensive characterized subclonal architecture of driver genes and investigated their clinical relevance in colorectal cancer. The results revealed that subclonal ANK1 mutations, clonal CASP8 mutations, and clonal SMAD2 mutations are independent risk factors for poor prognosis in CRC patients. Subclonal ANK1 mutation is related to adverse outcomes of CRC through increasing IL4I1, IDO1, IFNG and MAPK12, which may cause tumor immune escape through the accumulation of immunosuppressive cells such as regulatory T cells and myeloid derived suppressive cells. These results suggested that the clonality of driver genes could act as prognostic markers and potential therapeutic targets in human colorectal cancer.

## Material and methods

### Data source

The mutation profiles of colorectal cancer generated using whole-exome sequencing (WES) were obtained from The Cancer Genome Atlas network (TCGA) database. Copy number profiles of colorectal cancer derived from Affymetrix SNP 6.0 were obtained from TCGA. The RNA-seq FPKM data of colorectal cancer was accessed through TCGAbiolinks [9] to charaterize the transcriptional effect of clonality of driver genes. We obtained corresponding clinical metadata of colorectal cancer patients from the public cBio Cancer Genomics Portal (http://www.cbioportal.org) and microsatellite instability (MSI) information was acquired from Broad Institute Firehose (https://gdac.broadinstitute.org). Tumors originating from the splenic flexure, sigmoid colon, descending colon, or rectum was classified as left-sided and tumors originating from the appendix, ascending colon, cecum, hepatic flexure, or transverse colon was classified as right-sided. Among the 629 colorectal cancer patients, we mainly focused on 536 patients with both copy number aberrations, tumor purity, and clinical information. The clinicopathological characteristics were summarized in Additional file 1: Table S1.

### Inferring the cancer cell fraction and clonal status of somatic mutations in CRC patients

We apply ABSOLUTE algorithm [10] to infer tumor purity and absolute copy numbers by integrating WES and SNP array data from colorectal cancer. Following the framework previously proposed by McGranahan et al. [11] and Landau et al. [6], the cancer cell fraction (CCF) of each somatic mutation and the clonality of driver genes were identified through integrating tumor purity and local somatic copy number. Specifically, for each mutation site, the expected variant allele frequency (VAF) was dependent on the tumor purity, the local copy number of this mutation site, and the CCF of this mutation. Thus, the expected VAF could be calculated by giving a CCF according to the following equation:

$$VAF_{expected} = \frac{{tumor \, purity*CCF*C{\text{opy}}N{\text{um}}_{mutation} }}{{CPN_{{{\text{normal}}}} (1 - tumor \, purity) + tumor \, purity*CPN_{tumour} }}$$

where CopyNum_*mutation*_ was the mutation copy number in the cancer cell. CPN_*normal*_ and CPN_*tumor*_ denote the local copy numbers of the mutation site in normal and cancer cells, respectively. We let CPN_*normal*_ = 2 for autosome due to diploid of the human genome according to the study [11]. The somatic variants present at a single copy per cancer cell. As previous studies [11, 12], we assumed CopyNum_*mutation*_ to be 1 to avoid overcalling subclonality. For a given mutation with ‘t’ alternative reads, and a depth of ‘N’, the probability of a given CCF can be estimated using a binomial distribution:

$$P(CCF|(t|N)) \propto Binomial(t|N,VAF_{{_{expected} }} )$$

CCF values can then be calculated over a uniform grid of 100 CCF values (0.01,1) and subsequently normalized to obtain a posterior distribution [12]. We defined the P(subclonal) as the probability that a mutation is subclonal:

$$P(subclonal) = \Pr obability(CCF < 0.9)$$

Finally, mutations were classified as subclonal if the 95% confidence interval of the CCF cannot overlap 1 and probability that mutation has a cancer cell fraction less than 0.9 must be > 0.5 (i.e. P(subclonal) > 0.5), otherwise, the mutations were considered clonal.

### Clonal or subclonal mutation enrichment analysis

A permutation test was employed to assess whether a driver gene was enriched with clonal or subclonal mutations. Specifically, for a driver gene *D* harboring *n* non-silent mutations including *c1* clonal mutations and *s1* subclonal mutations across 536 CRC patients (c1 + s1 = n). The observed enrichment ratio of the driver gene Dg for clonal mutations and subclonal mutations could be denoted as clonal ratio = c1/s1 and subclonal ratio = s1/c1, respectively. We would randomly sample *n* non-silent mutations from background mutation sets of 536 CRC samples and calculated the random clonal ratio and subclonal ratio for gene *D*. After repeating the procedure 1000 times, we would obtain a *P*-value of clonal enrichment by dividing the times when the random clonal ratio was greater than the observed clonal ratio (*c1/s1*) by 1,000. The *P*-values of subclonal enrichment were estimated using the similar methods[13]. The false discovery rate (FDR) correction was assessed and reported along with the Q-value (FDR-adjusted *P*-value).

### Statistical methods

Categorical variables were compared by Fisher’s exact test. Continuous variables were compared by the Wilcoxon rank-sum test or a two-tailed unpaired Student’s t-test. Kolmogorov–Smirnov test was used to test for uniform distribution of CCFs of driver genes. The log-rank test was used to compare Kaplan–Meier curves. To test the independence of the prognostic value of the clonality of driver genes, we performed a comprehensive multivariate Cox analysis with stepwise elimination of non-significant covariates. All of the covariates in the Cox model satisfied the proportional hazard regression assumptions. Cox model stability was internally validated using bootstrapping procedures [14–16]. These approaches provided an estimate of the prediction accuracy of the cox model to protect against overfitting. Thresholds for CCFs with a good prediction of clinical outcome were calculated using Maximally selected rank statistics (maxstat R package). All statistical analyses were conducted using R software version 3.6.1 (http://www.rproject.org).

## Results

### Comprehensive characterization of clonality of driver genes in colorectal cancer

**Fig. 1:**
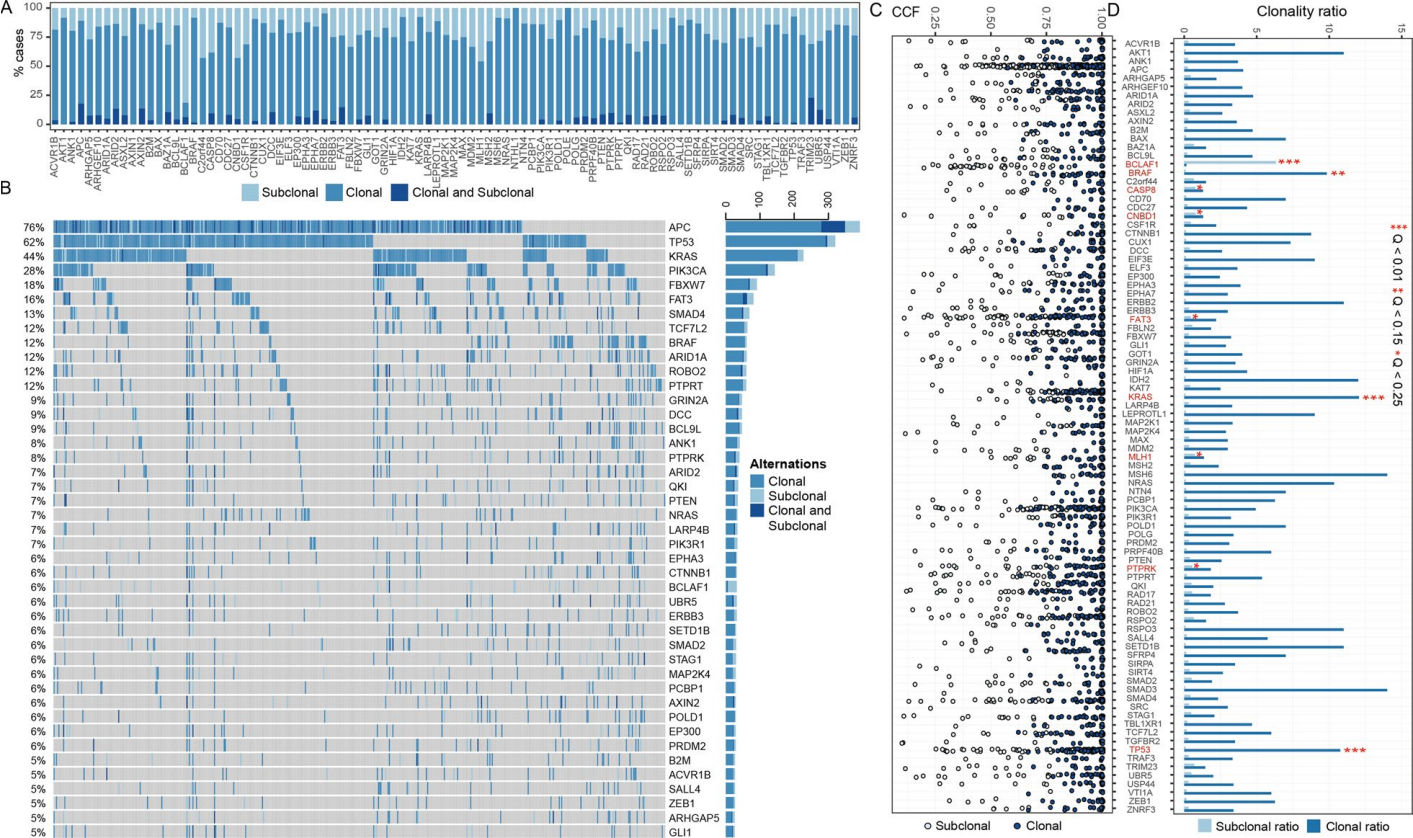
Comprehensive characterization of subclonal architecture of the 93 driver genes. **A** The percentage of patients carrying subclonal, clonal, and clonal-subclonal mutations in each gene. **B** The clonality of frequently altered genes in CRC. Each column represents a CRC case carrying at least 1 mutation in any of the studied genes. The bar plot on the right represents the number of times at which each mutation is observed in all mutated cases. **C** The distributions of cancer cell fraction (CCF) values for somatic events within CRC driver genes. The dark-blue dot represents clonal mutation for a driver gene. The pale-blue dot represents subclonal mutation for a driver gene. **D** Significant enrichment of CRC driver genes with clonal and subclonal mutations. The y-axis represents clonal ratio (c1/s1) and subclonal ratio (s1/c1) for each driver gene. c1 represents the number of clonal mutations in the driver gene; s1 represents the number of subclonal mutations in the driver gene. Q value (i.e. FDR-adjusted *P*-value) was obtained using the permutation test. The names of driver genes significantly enriched with clonal or subclonal alterations are labeled in red

In order to comprehensive characterize clonality of driver genes in colorectal cancer, we integrated mutation data, tumor purity and local absolute copy numbers to identify subclonal and clonal mutations (see Material and methods). Furthermore, we identified 97 colorectal cancer driver genes through integrating MutSigCV, MutSigCL, and MutSigFN tests to identify significantly mutated driver genes (FDR ≤ 0.1). We then characterized the subclonal architecture of genetic mutations in driver genes in CRC tumors (Fig. 1). As a result, 4167 non-silent driver mutations were identified in 95% (513/536) of CRC patients, including 3304 clonal mutations and 863 subclonal mutations in CRC driver genes. Clonal mutations occurred in 94.6% of patients, which were the sole mutations in 41.8% and associated with additional subclonal mutations in 52.8%. Solely subclonal mutations were found in 1.1% of the patients (Additional file 1: Fig. S1A). Specifically, we found that 4 driver genes (*RBM10, BCOR, DCAF12L2, ZMYM3*) without clonal and subclonal mutations and 4 driver genes (*AXIN, POLE, SMAD3, NTHL1*) only had clonal mutations in patients. We provided the landscape of the clonal and subclonal mutations of driver genes (Fig. 1A, 1B). According to gene mutation frequency, the top 10 clonal mutation genes were *TP53, APC, KRAS, PIK3CA, FBXW7, BRAF, TCF7L2, FAT3, ARID1A,* and *PTPRT* (Fig. 1B). The top 10 subclonal mutation genes were *APC, BCLAF1, TP53, FBXW7, PIK3CA, FAT3, SMAD4, KRAS, MLH1,* and *PTPRK.* Especially, *BLCAF1* was the unique gene whose subclonal mutations were less than clonal mutations. It was also observed that 54 genes simultaneously occur clonal mutations and subclonal mutations in the same patient, such as *APC, FAT3, PIK3CA, ARID2, DCC, ROBO2, TP53, AXIN2, FBXW7,* and *PTPRK*. Notably, clonal mutations were considered as a unique abnormality in almost all investigated driver genes (93/93, 100%) accounting for 18.75–100% of mutations per gene (median 80.95%) (Additional file 1: Fig. S1A). Similarly, All driver genes showed a non-uniform distribution of CCFs (Kolmogorov–Smirnov test, FDR < 0.05), and most mutations in all driver genes have CCF between 0.75 and 1 (Fig. 1C). We further observed that a clear tendency for indel mutations in *APC, CASP8, MLH1, PTEN,* and *TP53* to be subclonal compared to other mutation types. Nonsense mutations in *APC* and *PIK3R1* were enriched in clonal mutations when compared with other mutation types (Additional file 1: Fig. S1B, Table S2, Fisher’ exact test, P < 0.05).

We also provided the landscape of 43 frequently altered driver genes which mutated in at least 5% of samples in the TCGA CRC samples (Fig. 1B). Of these genes, *TP53* (P < 0.0001), *KRAS* (P < 0.0001) were significantly enriched with clonal non-silent mutations (Fig. 1C). These results were generally in accord with previous multi-region genome/exome sequencing of 24 benign and malignant colorectal tumors pointing out that mutations in *TP53* and *KRAS* tended to be truncal [17]. Also, some frequently studied genes in CRC showed a tendency to be enriched with clonal mutations or subclonal mutations. For example, Mutations in *BRAF* (P = 0.004), a gene highly associated with a variation on the usual CRC adenoma-carcinoma progression theme and the altered DNA-methylation phenotype known as CIMP [18], showed a tendency to be clonal. *MLH1*, a mismatch repair gene making MSI cases possess deficient DNA mismatch repair [19], had a higher number of subclonal mutations in CRC tumors (P = 0.01). Furthermore, several frequently mutated genes without well-established roles in CRC were also found to be enriched with subclonal mutations. For example, mutations in *BCLAF1* (P < 0.0001), *CNBD1* (P = 0.009), *PTPRK* (P = 0.015), *FAT3* (P = 0.005) and *CASP8* (P = 0.004) tended to be subclonal (Fig. 1D). As a result, these findings highlighted that clonal mutations and subclonal mutations in driver genes play a potential role in either the genesis or progression of CRC.

### Correlation between the clonality of driver genes and clinical parameters

**Fig. 2:**
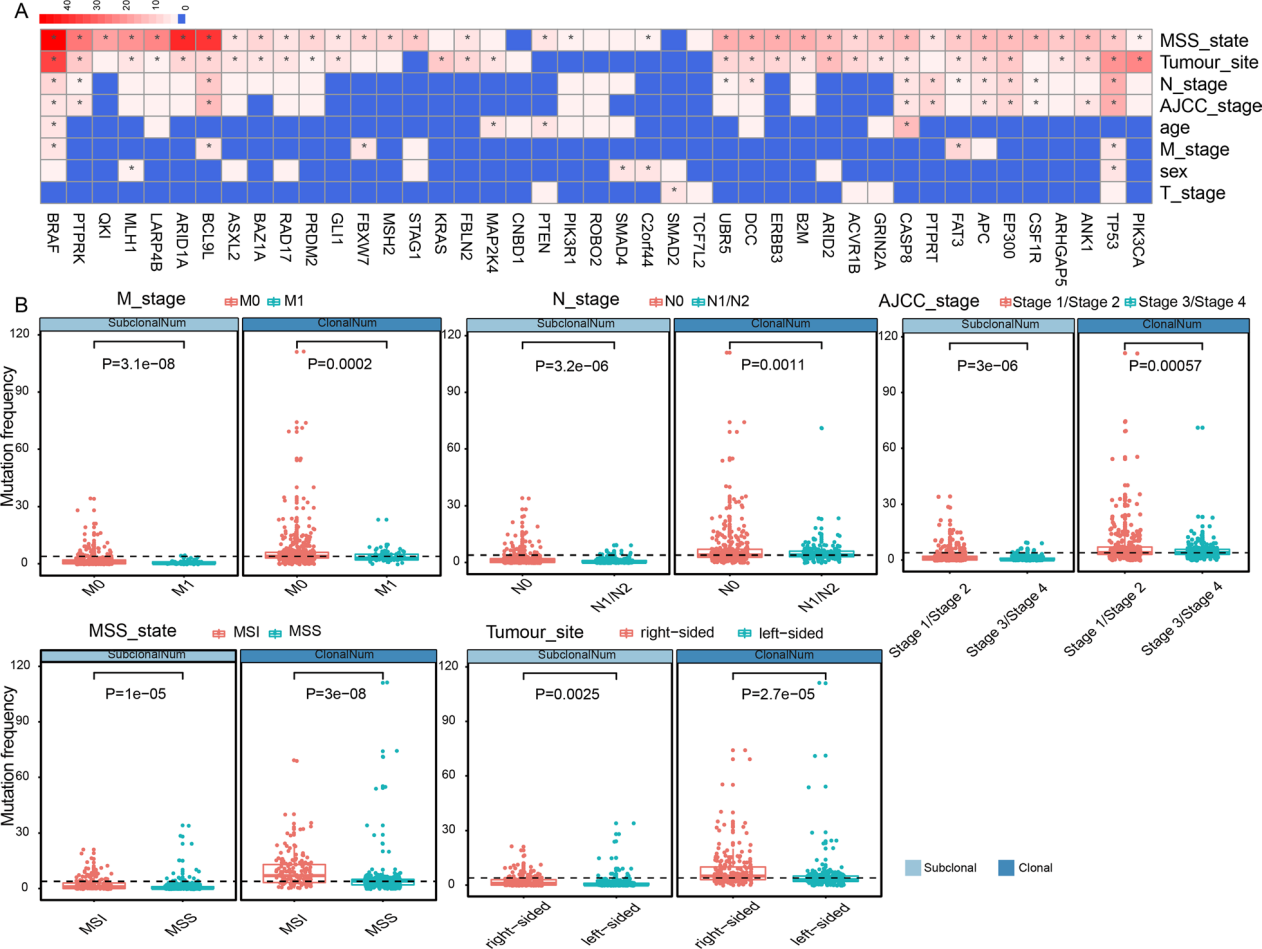
The significant correlations between the clonality of driver genes and clinicopathological characteristics. **A** The differences were performed using Fisher’s exact test (P values < 0.05). Heatmap is color-coded based on -log2(P-value). **B** Comparison of the number of subclonal and clonal mutations of patient grouped by clinicopathological factors, P-value is calculated by two-tailed unpaired Student’s t-test

We investigated whether there was an association between the clonality of driver genes and clinical parameters, such as microsatellite instability state, AJCC stage, TNM stage. Among 43 driver genes of which both clonal mutations and subclonal mutations occurred in at least 5 patients, the clonality of 40 driver genes (40/43) was significantly correlated with MSI status, tumor location, AJCC stage, N stage, M stage, T stage, sex, and age (Fisher’s exact test, P < 0.05, Fig. 2A). Furthermore, the correlation analysis showed that the clonal status of 4 genes (*TP53, SMAD4, C2orf44, MLH1*) was correlated with sex, and the *BRAF, CASP8, MAP2K4, PTEN* genes were related to age. And 10 genes (*APC, TP53, BRAF, CASP8, BCL9L, EP300, PTPRK, PTPRT, ANK1, CSF1R*) were discovered to be significantly related to the AJCC stage. In addition, We also found that 12 genes (*APC, TP53, BRAF, CASP8, FAT3, BCL9L, EP300, PTPRK, PTPRT, UBR5, CSF1R, DCC*) were significantly associated with N stage, the *SMAD2* gene was correlated with T stage and 5 genes (*TP53, FBXW7, BRAF, FAT3, BCL9L*) showed their significant association with M stage. Surprisingly, The clonal status of 36 genes accounting for 83.7% (36/43) of the remaining 43 driver genes was related to microsatellite instability state, and the clonal status of more than half of the remaining 43 driver genes show a tendency to be associated with tumor location. For example, clonal status of *TP53* mutation are significantly associated with sex, MSI status, tumour site, AJCC stage, N stage and M stage (Table 1). Clonal status of *BCL9L* mutation are significantly associated with MSI status, tumour site, AJCC stage, N stage and M stage (Table 2). In addition, we examined the association of clinicopathological factors with the prevalence of clonal and subclonal mutations in all 93 driver genes. MSI status and right-sided tumor location were found to be associated with greater number of clonal and subclonal mutations (MSI versus MSS, clonal: P < 0.001, subclonal: P < 0.001; right-sided versus left-sided, clonal: P < 0.001, subclonal: P < 0.05; Fig. 2B), but N_stage, M_stage and AJCC_stage were associated with less number of clonal and subclonal mutations (N1/N2 versus N0, clonal: P < 0.001, subclonal: P < 0.001; M1 versus M0, clonal: P < 0.001, subclonal: P < 0.001; stage3/stage4 versus stage1/stage2, clonal: P < 0.001, subclonal: P < 0.001; Fig. 2B). In summary, these findings implied that the clonality of driver genes had potentially important relationships with clinicopathological factors.

**Table 1:** Association between the clonal status of *TP53* mutation and clinical features in colorectal cancer

	Clonal	Subclonal	WT	P-value
Age				
< 67.5(median)	153	9	103	0.346
> = 67.5(median)	139	14	113	
Sex				
Female	129	17	108	0.015*
Male	163	6	108	
MSI status				
MSI	66	9	82	< 0.001*
MSS	225	14	133	
Tumour site				
Right-sided	92	11	117	< 0.001*
Left-sided	193	11	90	
AJCC stage				
Stage 1/Stage 2	134	16	141	< 0.001*
Stage 3/Stage 4	149	6	68	
N stage				
N0	142	17	147	< 0.001*
N1/N2	150	6	67	
T stage				
T1/T2	54	5	54	0.176
T3/T4	238	18	160	
M stage				
M0	206	15	169	0.016*
M1	50	0	22	

Significant P values are labeled with *(P < 0.05). WT: wide type

**Table 2:** Association between the clonal status of *BCL9L* mutation and clinical feartures in colorectal cancer

	Clonal	Subclonal	WT	P-value
Age				
< 67.5(median)	19	6	241	0.19
> = 67.5(median)	19	1	248	
Sex				
Female	17	3	237	0.89
Male	21	4	252	
MSI status				
MSI	30	5	121	< 0.001*
MSS	8	2	366	
Tumour site				
Right-sided	25	3	191	0.004*
Left-sided	12	4	281	
AJCC stage				
Stage 1/Stage 2	30	6	253	< 0.001*
Stage 3/Stage 4	7	0	219	
N stage				
N0	30	7	269	< 0.001*
N1/N2	8	0	218	
T stage				
T1/T2	6	3	104	0.26
T3/T4	32	4	383	
M stage				
M0	31	5	356	0.012*
M1	0	0	72	

Significant P values are labeled with *(P < 0.05)

### Prognostic impact of driver genes mutation clonality in colorectal cancer.

**Fig. 3:**
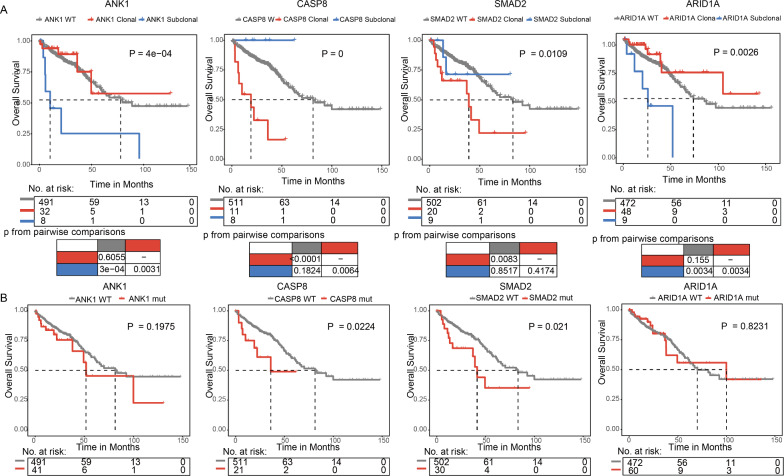
Survival analysis of driver genes in the TCGA colorectal cohort. **A** Comparison of overall survival among patients carrying clonal mutations (red line), subclonal mutations (blue line), and patients harboring unmutated genes (gray line) by Kaplan–Meier analysis (with log-rank values) in the cohort of colorectal cancer patients from TCGA. **B** Kaplan–Meier survival analysis of driver genes stratified by the mutation status of driver genes

To determine whether the clonality of the mutations may influence the clinical outcome of the patients, we analyzed above 43 driver genes with clonal and subclonal mutations in at least 5 patients. And the Kaplan–Meier curve analysis suggested that 4 genes (*ANK1, CASP8, SMAD2, ARID1A*) were found to be associated with a prognostic value (Fig. 3A). Patients with subclonal ANK1 mutations tended to have a reduced median overall survival than patients with clonal *ANK1* mutations (log-rank test, P = 0.0031) and patients with *ANK1* wide type (log-rank test, P = 3e-04) with 5-year OS at 21.4% (subclonal), 55.3% (clonal) and 61.0% (wide type) respectively. However, there is no prognostic significance of the mutation status of *ANK1* (P = 0.20; Fig. 3B). Patients with subclonal *ARID1A* mutations tended to have a reduced overall survival than patients with clonal *ARID1A* mutations and patients with *ARID1A* wide type (P = 0.0034; Fig. 3A). However, there is no prognostic significance of the mutation status of *ARID1A* (P = 0.82; Fig. 3B). When the survival outcomes were analyzed using *CASP8*, overall survival was significantly shorter in patients with clonal mutations than patients with subclonal mutations (log-rank test, P = 0.0064) and than wide type cases (log-rank test, P < 0.0001) with 3-year OS at 16.4% (clonal), 100% (subclonal) and 79.5% (wide type) respectively. Whereas patients harboring clonal *SMAD2* mutations had a trend toward a shorter overall survival compared with patients harboring wide type sequence (log-rank test P = 0.0083), with no significant difference between clonal and subclonal mutations with 5-year OS 22.1% (clonal) versus 71.4% (subclonal) versus 61.6% (wide type). The subclonal architecture of *CASP8* (P = 0) and *SMAD2* (P = 0.011) could increase the prognostic value compared to the mutation status of *CASP8* (P = 0.022) and *SMAD2* (P = 0.021), respectively (Fig. 3B). Several mutated genes with prognostic impact were significantly co-occurring in the same patient (Additional file 1: Fig. S2). To identify which of them had an independent prognostic value, we performed univariate, multivariable, and backward-stepwise cox regression analysis including the mutated genes with prognostic impact in the KM analysis together with microsatellite instability state and clinical parameters (gender, age, MSI_status, T_stage, N_stage, M_stage, Tumour_location, TNM_stage). These analysis revealed that subclonal *ANK1* mutations (HR = 6.09, CI = 1.36–27.29, P = 0.018), clonal *CASP8* mutations (HR = 5.91, CI = 2.43–14.36, P = 0.0001), and clonal *SMAD2* mutations (HR = 3.51, CI = 1.6388–7.55, P = 0.001) are independent risk factors for poor prognosis (Table 3). Clinical factors including age, M_stage, and TNM_stage were also independently associated with a shorter overall survival of patients. Consistently, the maximally selected rank statistics succeeded to identify appropriate cut-off points capable of best predicting CRC overall survival in two genes: *SMAD2* (cutoff = 0.79, P = 0.014) and *CASP8* (cutoff = 0.78, P = 0.001). The internal validity of the model was evaluated using bootstrapping and 3 genes (*CASP8*, *ANK1*, *SMAD2*) were selected for the model in 88% of 1000 repeats (Additional file 1: Table S3).

**Table 3:** Univariate and multivariate analysis of the prognostic impact of clinicopathological variables and the clonality of driver genes

	Univariate analysis			Multivariate analysis			Stepwise analysis			Bootstrap		
	HR	CI	P	HR	CI	P	HR	CI	P	HR (mean)	CI (mean)	Inclusion frequency
ANK1												88%
Subclonal vs WT	4.53	1.97–10.43	< .001	6.09	1.36–27.29	0.018	6.64	1.6–27.5	9e−3	7.02	1.78–28.84	
Clonal vs WT	0.78	0.32–1.93	0.60	0.95	0.31–2.95	0.932	0.99	0.32–3.07	0.98	1.06	0.34–3.55	
ARID1A												64.9%
Subclonal vs WT	3.68	1.49–9.07	4.6e−3	1.20	0.22–6.70	0.83	1.66	0.32–8.54	9e−3	2.62	0.45–29.3	
Clonal vs WT	0.58	0.27–1.24	0.1581	0.18	0.06–0.60	0.005	0.23	0.07–0.71	1.1e−2	0.21	0.07–0.65	
SMAD2												88.8%
Subclonal vs WT	1.14	0.28–4.63	0.85	2.90	0.37–22.46	0.31	2.07	0.28–15.5	0.48	4.42	0.96–22.80	
Clonal vs WT	2.61	1.36–4.999	0.0038	3.51	1.639–7.55	0.001	3.76	1.78–7.91	1e−3	4.45	2.03–9.93	
CASP8												92.5%
Subclonal vs WT	0	0-Inf	0.993	0	0-Inf	0.994	0	0-inf	0.995	0.025	0-inf	
Clonal vs WT	5.28	2.56–10.88	< 1e−4	5.91	2.43–14.36	1e−4	6.33	2.63–15.23	< 1e−3	7.17	2.82–18.82	
Age												100%
> = 67.5 vs < 67.5	2.14	1.44–3.17	2e−04	3.25	1.99–5.32	< 1e−4	3.04	1.89–4.89	< .001	3.59	2.16–6.01	
Sex												36.1%
Male vs Female	1.05	0.73–1.52	0.79	0.78	0.51–1.20	0.26	–	–	–	0.65	0.41–1.02	
Tumour site												48.5%
Left vs Right	0.72	0.49–1.04	0.08	0.74	0.48–1.15	0.186	0.7	0.45–1.07	0.097	0.60	0.38–0.95	
M_stage												98.2%
M1 vs M0	3.92	2.56–5.99	< 1e−4	2.92	1.68–5.07	1e−4	3.22	1.89–5.49	< .001	3.19	1.82–5.59	
N_stage												45.7%
N1/N2 vs N0	2.47	1.70–3.59	< 1e−4	0.53	0.17–1.68	0.28	–	–	–	0.94	0.49–1.90	
T_stage												39.3%
T3/T4 vs T1/T2	2.46	1.28–4.72	0.006	1.76	0.72–4.34	0.22	–	–	–	3.09	1.00–10.07	
TNM_stage												88.7%
Stage 3/4 vs Stage 1/2	2.70	1.82–4.01	< 1e−4	5.31	1.49–19.02	0.01	2.78	1.62–4.76	< .001	5.01	2.16–12.91	
MSI status												34.1%
MSI vs MSS	0.80	0.54–1.15	0.22	0.79	0.48–1.29	0.34	–	–	–	0.63	0.38–1.06	

Next, subgroup analyses evaluating the prognostic effect of clonality of driver genes were performed for stratifications by staging, tumor location, and MSI status. Among the AJCC stage I and stage II patients (Additional file 1: Fig. S3A), patients with clonal *CASP8* mutations showed significantly shorter overall survival as compared to patients with subclonal *CASP8* mutations (P = 0.0044) and wide type cases (P < 0.0001) with 5-year overall survival at 16.7% (clonal), 100% (subclonal) and 92.8% (wide type) respectively. However, patients harboring clonal *SMAD2* mutations had a trend toward a shorter overall survival compared to the patients with *SMAD2* wide type (P = 6.0e−4), with no significant difference between clonal and subclonal mutations (P = 0.5641) with 5-year overall survival 25% (clonal) versus 75% (subclonal) versus 82.4% (wide type). Furthermore, compared with patients with clonal *ANK1* mutations (P = 0.0271) and wide type cases (P = 0.0037), the patients with subclonal *ANK1* mutations significantly predicted shorter overall survival with 5-year overall survival 85% (clonal) versus 30% (subclonal) versus 79.2% (wide type). When stratified by MSI status (Additional file 1: Fig. S3B), overall survival was significantly shorter in MSI patients with subclonal *ARID1A* mutations as compared to MSI patients with clonal *ARID1A* mutations (P = 0.0194) and wide type cases (P = 0.0194) with 3-year overall survival at 43.8% (subclonal), 83.1% (clonal) and 74.5% (wide type) respectively. In addition, MSI subgroup harboring subclonal *ANK1* mutations had significantly shorter overall survival than MSI subgroup with WT sequence (P = 0.03), and no significant differences were observed between clonal *ANK1* mutations and subclonal *ANK1* mutations within MSI subgroup with 3-year overall survival 82.5% (clonal) versus 25% (subclonal) versus 75.9% (wide type). Furthermore, we also observed that overall survival was significantly shorter in patients with clonal *CASP8* mutations than patients harboring subclonal *CASP8* mutations (log-rank test P = 0.0421) and WT cases (log-rank test P = 0.007) with 3-year overall survival 31.2% (clonal) versus 100% (subclonal) versus 76.2% (wide type). For right-sided subgroup (Additional file 1: Fig. S3C), patients with subclonal *ANK1* mutations or subclonal *ARID1A* mutations significantly predicted shorter overall survival than those with clonal mutations (P = 0.0046 and P = 0.0071, respectivily) and wide type cases (P < 0.0001 and P = 0.0322, respectivily), with no significant difference between patients with clonal mutations and wide type cases. These results indicated that clonal or subclonal mutations of driver genes are independent prognostic factor for survival in patients with CRC.

### Subclonal *ANK1* mutation-driven transcriptional effect is associated with the level of immune cell infiltration in CRC

We sought to explore the potential molecular mechanism of subclonal *ANK1* mutations which has been associated with poor prognosis in CRC. Differentially expressed genes (DEGs) were identified between CRC patients with and without *ANK1* mutation as ANK1-mutant gene signatures using DESeq2 [20] and EdgeR [21] (Additional file 1: Fig. S4A, B; FDR < 0.05, fold change > 2). A total of 139 upregulated genes and 149 downregulated genes were identified as *ANK1*-mutant gene signature. Then, single-sample gene set enrichment analysis (ssGSEA) using the GSVA [22] was performed and showed that *ANK1*-mutant upregulated genes are significantly enriched in patients with subclonal *ANK1* mutation than clonal *ANK1* mutation group (P = 0.017, Additional file 1: Fig. S4C). The *ANK1*-mutant downregulated genes are significantly enriched in patients with clonal *ANK1* mutation than subclonal *ANK1* mutation group (P = 0.036, Additional file 1: Fig. S4C). The negative prognostic impact was highly specific to patients with subclonal *ANK1* mutations (log-rank test P = 0.0031, subclonal HR = 4.53; P < 0.001 versus clonal HR = 0.78; P = 0.60; Fig. 3), reinforcing a clinical relevance of the stronger transcriptional effect of the subclonal *ANK1* mutations in this population.

**Fig. 4:**
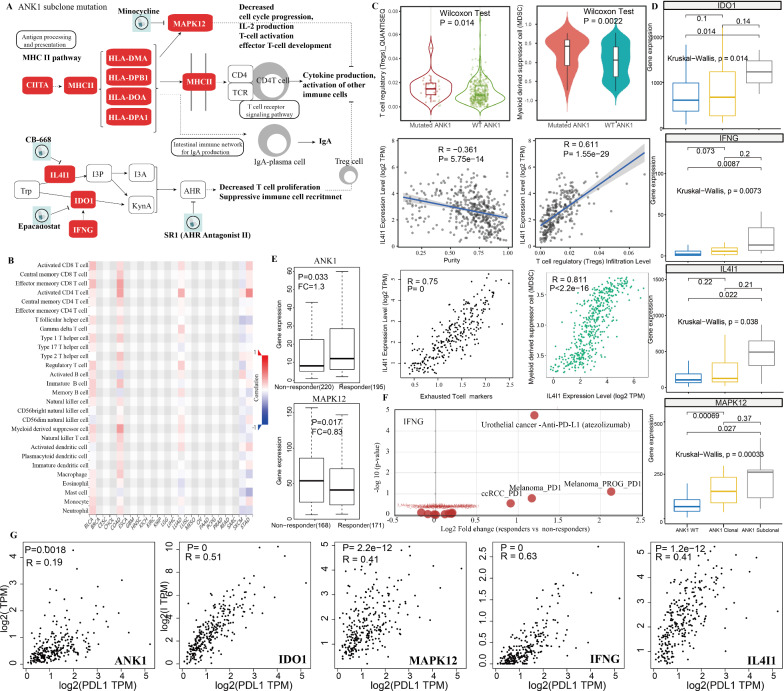
Subclonal ANK1 mutation-driven transcriptional effect is associated with the level of immune cell infiltration in CRC. **A** ANK1 subclonal mutation-driven genes affect multiple important biological pathways. **B**, **C** Spearman correlations between of ANK1 subclonal mutation, ANK1-driven genes and immune cells across human using TISIDB, TIMER and GEPIA. **D** Differential expression of IDO1, IGNG, IL4I1 and MAPK12 among CRC patients with clonal ANK1 mutation (yellow), subclonal ANK1 mutation (gray), and ANK1 wide type (blue). **E** Expression levels of ANK1 and MAPK12 in CRC patients in relation to response to chemotherapy treatment. Patients were classified as responder or nonresponder according to the 5-year relapse-free survival. P-value is calculated using Mann–Whitney test. **F** The significant difference of INFG expression between responders (68 samples) and non-responders (230 samples) in urothelial cancer patient cohorts with PD-L1 blockade cancer immunotherapy. **G** correlation of PD-L1 and ANK1-driven genes in colon cancer types

Specifically, upregulated gene signatures in subclonal ANK1 mutations were significantly enriched in antigen processing and presentation (FDR = 9.07e−4), intestinal immune network for IgA production (FDR = 0.007), Inflammatory bowel disease (FDR = 0.002), and cell adhesion molecules (CAMs; FDR = 0.01). For example, ANK1 subclonal mutation-driven major histocompatibility complex (MHC) class II (including HLA-DMA, HLA-DPA1, HLA-DOA, and HLA-DPB1) and MHC class II transactivator CIITA are increased in CRC patients with subclonal ANK1 mutations which mediated processing and presentation pathway and needed for activation of T cell and other immune cells [23] (Fig. 4A and Additional file 1: Fig. S4D). We investigated the relationship between mutation of ANK1, ANK1-driven genes and immune markers of tumor-infiltrating immune cells such as T cells, B cells, macrophages, neutrophils, dendritic cells and different subsets of effector T cells in TCGA colorectal cancer using gene set variation analysis (GSVA) and Spearman's correlation analysis. We found that mutation of ANK1 positively correlated with infiltration levels of myeloid derived suppressor cells (MDSC; Wilcoxon test P = 2.2e−3), regulatory T cells (Tregs; P = 0.014), effector memeory CD8 T cells (P = 6.08e−4) and activated CD4 T cells (P = 6.23e−4; Fig. 4B, 4C and Additional file 1: Fig. S5). Subclonal ANK1 mutations-driven genes HLA-DMA, HLA-DPB1 HLA-DPA1, and HLA-DOA show positive associations with infiltration levels of CD4 + T cell, CD8 + T cell and regulatory T cells (Additional file 1: Fig. S6). Subclonal ANK1 mutations could increase MAPK12 expression (Fig. 4D) which show negative associations with infiltration levels of CD4 + T memory cell and B cell plasma (Additional file 1: Fig. S5). We obtaind gene expression datasets of 440 colorectal cancer patients with chemotherapy agents, such as 5-fluorouracil and oxaliplatin, and response data from Balázs Győrffy et al. [24]. We found that significant differences in ANK1 and MAPK12 expression were detected between responders (no resid ualhistological evidence of tumor) and non-responders (with residual tumor tissue) after chemotherapy (Mann–Whitney test, FC = 1.3, P = 0.017 for ANK1; FC = 0.83, P = 0.033 for MAPK12), suggesting their correlation to chemotherapy resistance in colorectal cancer.

Most importantly, interleukin-4-induced-1 (IL4I1) and indoleamine 2 3-dioxygenase 1 (IDO1) are upregulated in subclonal ANK1 mutation CRC patients (Fig. 4D), which could inhibit T cell activation by inhibition of effector cell proliferation and immunoregulation mediated by Treg cells induction [25]. IL4I1 could activate the AHR through I3P-KynA/I3A metabolic pathway parallel to IDO1-driven AHR signaling which together suppresses T cell proliferation and function (Fig. 4A). A recent study showed a strongly association between high IL4I1 levels and an accumulation of myeloid-derived suppressor cells and Tregs in lymphocytic leukemia (CLL). Immune checkpoint blockade (ICB) could induce IL4I1 and IDO1, whereas IL4I1-deficient mice model showed a reduced Treg frequency and tumor burden, indicating the role of IL4I1 in immune escape [26]. Indeed, we found that the expression of IL4I1 shows positive associations with the abundance of Treg cells (Spearman correlation test: R = 0.611, P = 1.55e−29), myeloid derived suppressor cells (R = 0.811, P < 2.e−16) and exhausted T cell (R = 0.75, P = 0) and negative association with tumor purity (R = -0.361, P = 5.75e−14; Fig. 4C), indicated its immunosuppressive effects and promising target for CRC cancer immunotherapy. The above findings are generally consistent with previous research that ICB could induce the expression of IL4I1 and IDO1 [27]. Since IDO1 inhibitors such as epacadostat cannot block the expression of IL4I1, IL4I1 may be the reason for the failure of clinical studies on ICB combined with IDO1 inhibition [27], suggest that IL4I1 blocking (such as CB-668) combined with IDO1 and immune checkpoint suppression may be a candidate pathway for colorectal cancer therapy. Besides, subclonal ANK1 mutations could increase the expression of IDO1 and IFNG (Fig. 4D) which also show positive associations with infiltration levels of Treg cells (Spearman correlation test: R = 0.44 for IFNG, R = 0.586 for IDO1; both P < 2.2e−16), myeloid derived suppressor cells (R = 0.533 for IFNG, R = 0.649 for IDO1, both P < 2.2e−16; Additional file 1: Fig. S5). Using 298 urothelial cancer patient cohorts with PD-L1 blockade cancer immunotherapy with atezolizumab, we found significant upregulation of INFG expression between responders and non-responders (fold change = 2.3, Moderated T test P = 1.8e−05; Fig. 4F). We also found that ANK1 (P = 0.0018), IL4I1 (P = 1.2e−12), IDO1 (P = 0), IFNG (P = 0)and MAPK12 (P = 2.2e−12; Fig. 4G) are positively correlated with PD-L1 expression in CRC cancer which has been reported to be associated with worse prognosis and counteract effect of tumor-infiltrating lymphocytes [28, 29]. These results implicated that the upregulation of IL4I1, IDO1, IFNG and MAPK12 caused by subclone ANK1 mutations in CRC may lead to a accumulation of Treg cells and myeloid derived suppressor cells and thus a poor outcome in colorectal cancer. Taken together, our results showed that the significant correlation between subclonal ANK1 mutation, ANK1-driven genes and immune cell infiltration in CRC, suggesting subclonal ANK1 mutation as a prognostic marker plays potential role in immunotherapy for CRC.

### The pan-cancer analysis of clonality of driver genes

**Fig. 5:**
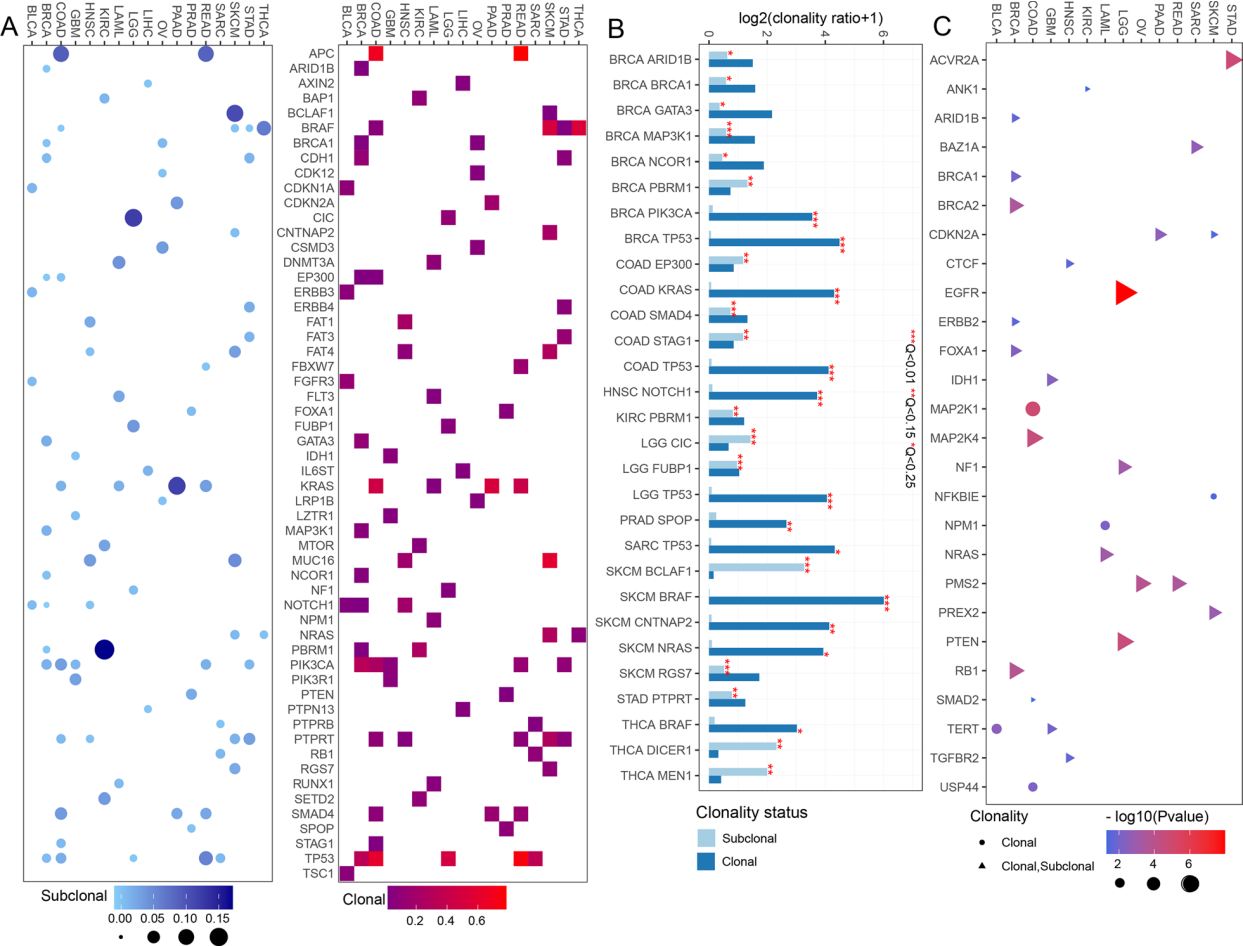
The pan-cancer analysis of clonality of driver genes. **A** The fraction of samples of each cancer type with subclonal (blue circles; left panel) and clonal (red squares; right panel). Drive genes with 5 or more mutations are shown. **B** Significant enrichment of driver genes with clonal and subclonal mutations. The names of driver genes significantly enriched with clonal (dark blue) or subclonal (light blue) alterations in each cancer are labeled in red stars. **C** Survival analysis of driver genes with clonal or subclonal mutations in at least two patient. Circles and triangles represent driver genes with clonal mutations and those with both types of mutations, respectively

In order to comprehensively characterize clonality of driver genes at the level of pan-cancer, the approach described in Method was applied to other eighteen TCGA cohorts by integrating the WES and SNP array data. We obtained 439 tumor-specific driver genes involving 18 cancer types from Cancer Genomics Consortium (CGC) database, with an average of 37 driver genes per cancer type. We identified the clonality of driver genes in 18 cancer types (Fig. 5A). As a result, of 11,517 driver mutations in 308 genes, we found 2,123 (18.4%) subclonal events in 238 driver genes and 9,394 (81.6%) clonal events in 276 driver genes. By analysis, 87.2% (3,660 of 4195) of analyzed samples have a clonal driver, 21.3% (895 of 4195) have not only clonal drivers but also subclonal drivers, and 12.7% (535 of 4195) have exclusively subclonal drivers. Mutations of specific driver genes is distinctive across cancer types. For example, tumor suppressor gene CIC was found to be enriched with subclonal mutations in lower grade glioma (LGG), as observed previously in WES and SNP array analysis (Fig. 5B) [30]. The subclonal mutation of tumor suppressor gene PBRM1 mainly occurred in renal clear cell carcinoma. Tumor suppressor gene CNTNAP2 has frequently clonal mutation in melanoma. Interestingly, mutations in some driver genes are exclusively clonal or subclonal in most cancer types. For example, we found that clonal mutations in TP53 are common in lower grade glioma, breast, colon, sarcoma and rectal cancer. Especially, TP53 is significantly enriched in clonal mutations in breast cancer, colon cancer and low grade glioma, which is consistent with the results that TP53 somatic mutations had a clonal advantage over other genes in breast cancer [31]. BRAF is commonly associated with clonal mutations in colon, stomach, melanoma and thyroid cancers, especially in melanoma and thyroid cancers. In addition, PBRM1 has significant subclonal mutations in breast cancer and kidney clear cell carcinoma.

Next, to determine whether clonal status of driver genes affect clinical outcomes in cancer patients, we used the approach described in Method to identify prognostic driver genes in 14 cancer types. We identified the clonality of 26 driver genes significantly associated with prognosis in fourteen cancer types, with an average of two driver genes per cancer type (Fig. 5C, Additional file 1: Table S4). We found that 11.5% (3/26) of driver genes including PMS2, CDKN2A and TERT are significant in the prognosis at least two cancer types. In particular, both clonal and subclonal mutations of TERT are associated with poor prognosis of bladder cancer and glioblastoma (*P*-value = 0.003 and *P*-value = 0.011, respectively). The findings are consistent with previous research that TERT mutations have been recognized as a common genetic event and show a high risk of recurrence in bladder cancer and GBM [32, 33]. Subclonal mutations in PMS2 are associated with adverse outcomes in rectal and ovarian cancer, while patients with clonal mutations have survival benefits (*P*-value = 3e−04 and *P*-value = 1.2e−04, respectively). The clonality of 88.6% (23/26) of driver genes only is significantly associated with the prognosis of a specific cancer type. For example, clonal and subclonal mutations of oncogene EGFR have a worse prognosis in LGG (*P*-value = 7.4e−14). This result is consistent with the recent report that EGFR mutation leads to an up-regulation of immune response related pathways and poor prognosis in LGG [34]. Clonal mutations of MAP2K1 are associated with a worse prognosis in colon cancer patients. These results suggested that clonal or subclonal mutations of driver genes play a potential role in the development or progression of cancer and are independent prognostic factors for cancer survival.

## Discussion

Genomic studies in cancers have recently characterized the complex heterogeneity of these diseases. The characterization of the subclonal architecture revealed the complexity of the mutational components of the genome. Recent studies have also emphasized a large number of driver genes and the influence of their subclonal heterogeneity in the outcome of the patients. Therefore, we comprehensive characterized clonality of driver genes and investigated their clinical relevance in colorectal cancer.

Our results suggest the presence of clonal heterogeneity in driver genes, including hotspot mutations, such as *APC*, *TP53*, and *KRAS*. We also found that the number of clonal mutations was much more than that of subclonal mutations in CRC driver genes, indicating that the majority of CRC driver genes are the basis of the tumorigenesis of cancer and tend to exist in the trunk of the phylogenetic tree during the cancer evolutionary process. Previous research has also emphasized the result in many cancer types [35, 36]. For example, Swanton et al. revealed that 219 of 795 driver events were found to be subclonal and 576 to be clonal in non-small-cell lung cancer. Edwin et al. also found that only 6.6% of all SNVs, MNVs, and indels across pan-cancer and just 3.7% of the point-mutation drivers were found to be subclonal. These results highlight the complex process of tumor evolution.

In this study, we demonstrated that the mutation clonality of *ANK1*, *CASP8*, *SMAD2,* and *ARID1A* had a significant impact on CRC patients' outcomes. Specifically, subclonal mutations of *ANK1* and *ARID1A* and clonal mutations of *CASP8* and *SMAD2* could predict shorter overall survival compared to patients with wild type sequence. Specifically, subclonal mutations of *ANK1* and *ARID1A* also predicted shorter overall survival compared to their corresponding clonal mutations. We observed that mutations in *ANK1* and *ARID1A* tend to occur together (Additional file 1: Fig. S4). Furthermore, when other clinical parameters were adjusted in multiple regression analysis by considering them as independent covariates, the subclonal *ANK1* mutations, clonal *CASP8* mutations and clonal *SMAD2* mutations could be independent prognostic factors for CRC. Indeed, some studies showed that the deletion of the long arm of chromosome 18 (loss of 18q or LOH of 18q) is the most common cytogenetic abnormality in CRC and seems to be associated with poor prognosis as 18q contains several important tumor suppressor genes, such as *SMAD2*, *SMAD4*, and *SMAD7* that are transcriptional mediators in the TGF-β signaling pathway [37]. Subgroup analysis suggested that the mutation clonality of *CASP8*, *ANK1*, *SMAD2,* and *ARID1A* showed significantly prognostic value among different CRC groups including MSI subgroup, AJCC stage I/II subgroup, and right-sided subgroup. Importantly, we found that subclonal ANK1 mutations could increase expression of IL4I1, IDO1, IFNG, MAPK12, CIITA and MHC class II which play important roles in antigen processing and presentation and immune response. Subclonal ANK1 mutation-drive genes show positive associations with infiltration levels of Treg cells and myeloid derived suppressor cells, which may cause tumor immune escape thus contributing to a poor outcome in colorectal cancer. Our results underlined the significant correlation between subclonal ANK1 mutation, ANK1-driven genes and immune cell infiltration in CRC, suggesting subclonal ANK1 mutation as a prognostic marker plays potential role in immunotherapy for CRC.

To further validate our results in independent datasets, we obtained somatic mutation, copy number variation and associated clinical data of 282 colorectal cancer samples derived from MSK-IMPACT targeted sequencing data from cBioPortal for Cancer Genomics [38]. In MSK-IMPACT cohort, 385 genes and 2545 mutation sites were detected. The approach described in Method was applied to MSK-IMPACT cohort to identified the clonality of mutations. In addition, we also used ABSOLUTE to estimate the cancer cell fraction (CCF) and classified a mutation as clonal if the CCF harboring it was > 0.95 with probability > 0.5 and subclonal otherwise. Comparison of these two methods in the MSK-IMPACT cohort showed 72.4% (1843/2545) agreement in clonality status of somatic mutation (*P*-value < 0.05; hypergeometric test) and an average 99.4% concordance in the clonality of 385 mutated genes (Additional file 1: Fig. S7A). In addition, we evaluated the proportion of clonal and subclonal mutations on common 47 CRC driver genes in the TCGA corhort and MSK-IMPACT cohort. We found that the fractions of clonal or subclonal mutations within driver genes were generally similar between the two cohorts, both of which showed a higher proportion of clonal mutations in the most of driver genes (Additional file 1: Fig. S7B). Furthermore, we next sought to characterize the association between the clonality of driver genes and clinical outcome using MSK-IMPACT cohort. The Kaplan–Meier curve analysis suggested that 6 genes (*GLI1*, *CASP8, EPHA7*, *POLE*, *TCF7L2* and *MSH6*) were found to be associated with a prognostic value using MSK-IMPACT cohort (Additional file 1: Fig. S7C). Three (*CASP8, SMAD2, ARID1A*) of the four prognostic genes identified in the TCGA dataset were detected in the MSK-IMPACT cohort. Overall, both of TCGA cohort and MSK-IMPACT cohort consistently identified the clonal mutation of *CASP8* as promising biomarkers to predict a poor prognosis in CRC patients.

In summary, we present a systematical characterization of the clonality of driver gene mutations and their clinical relevance in colorectal cancer. Our results underlined a significant association between the outcome of colorectal cancer patients and the clonality of driver genes, suggesting that the clonality of driver genes could act as prognostic markers and potential therapeutic targets in human colorectal cancer.

## Supplementary Information

**Additional file 1: Fig. S1**. Deep characterization of the clonal architecture of the 93 driver genes. **Fig. S2**. Heatmap of the co-occurrence of the driver mutations. **Fig. S3**. Subgroup analysis for CRC patients according to the TNM stage, MSI status, and tumor location. **Fig. S4**. ANK1-mutant gene expression signature. **Fig. S5**. Correlations between of ANK1, ANK1-driven genes IDO1, IFNG, MAPK12 and immune cells. **Fig. S6**. Correlations between of ANK1 subclonal mutation-driven major histocompatibility complex (MHC) class II and immune cells. **Fig. S7**. Assessing the consistency of clonality status of driver genes in the validation dataset. **Table S1**. Clinical and pathological parameters of patients in TCGA CRC cohort. **Table S2**. The associations between the clonality of driver genes and the mutation type. **Table S3**. Univariate and multivariate analysis of mutation status of driver genes. **Table S4**. Univariate and multivariate and backward-stepwise cox of the prognostic impact of clinicopathological variables and the clonality of driver genes in pan-cancer.

## Data Availability

All codes in this study are available in GitHub repository (https://github.com/shijianasdf/DrgsClonalityClinical).
