# Supplementary material for: Comprehensive characterization of clonality of driver genes revealing their clinical relevance in colorectal cancer
Source: J Transl Med. 2022 Aug 12;20:362. doi: 10.1186/s12967-022-03529-x (PMC9373375; doi:10.1186/s12967-022-03529-x)
Supplement: Supplementary file 1 — Additional file 1: Fig. S1. Deep characterization of the clonal architecture of the 93 driver genes. Fig. S2. Heatmap of the co-occurrence of the driver mutations. Fig. S3. Subgroup analysis for CRC patients according to the TNM stage, MSI status, and tumor location. Fig. S4. ANK1-mutant gene expression signature. Fig. S5. Correlations between of ANK1, ANK1-driven genes IDO1, IFNG, MAPK12 and immune cells. Fig. S6. Correlations between of ANK1 subclonal mutation-driven major histocompatibility complex (MHC) class II and immune cells. Fig. S7. Assessing the consistency of clonality status of driver genes in the validation dataset. Table S1. Clinical and pathological parameters of patients in TCGA CRC cohort. Table S2. The associations between the clonality of driver genes and the mutation type. Table S3. Univariate and multivariate analysis of mutation status of driver genes. Table S4. Univariate and multivariate and backward-stepwise cox of the prognostic impact of clinicopathological variables and the clonality of driver genes in pan-cancer. [file 12967_2022_3529_MOESM1_ESM.pdf]

SUPPLEMENTARY INFORMATION

SUPPLEMENTARY FIGURES

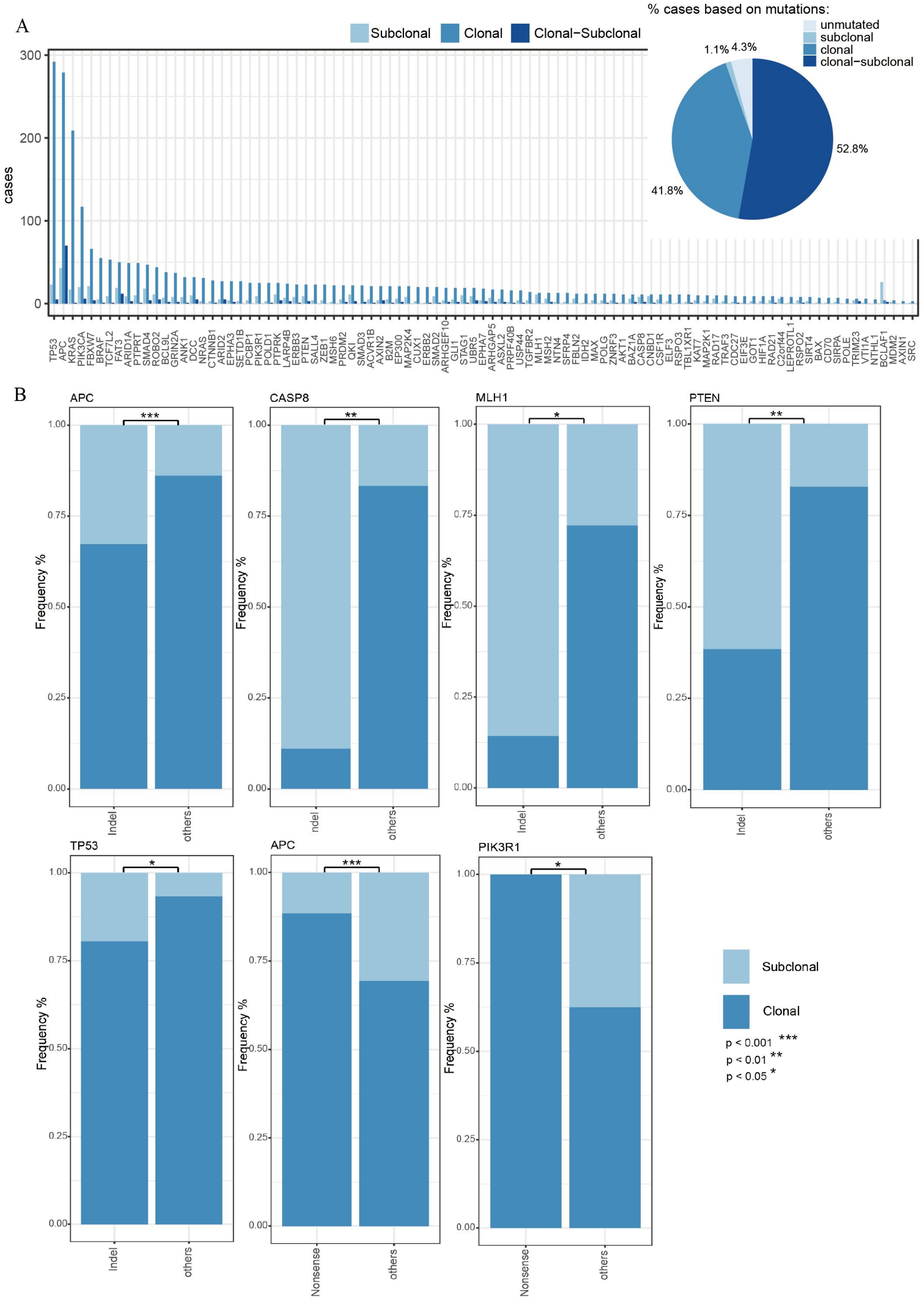

Supplementary Fig. 1. Deep characterization of the clonal architecture of the 93 driver genes.

(a) Pie chart of the proportion of patients grouped according to their mutational clonality in the entire cohort of 536 patients (top-right corner). The number of patients carrying subclonal, clonal, and clonal-subclonal mutations in each gene. (b) The proportion of Indel (Nonsense) versus other mutation types that are clonal/subclonal is indicated for each driver gene. Dark blue represents clonal mutations, and light blue represents subclonal mutations. P-value was calculated from Fisher's exact test.

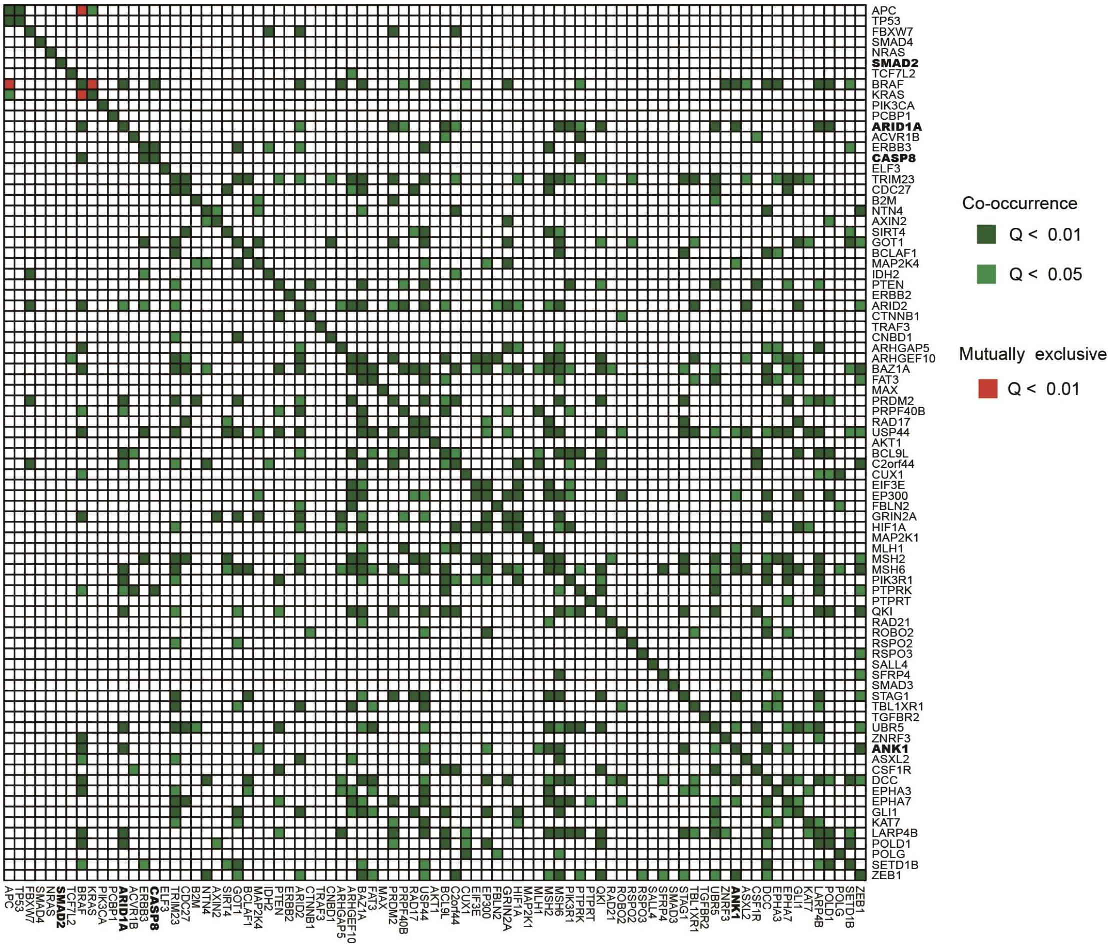

Supplementary Fig. 2. Heatmap of the co-occurrence of the driver mutations identified in ⩾ 10 patients by representing the adjusted P-value of the Fisher's exact test. Genes with prognostic value in the Kaplan–Meier analysis are depicted in bold.

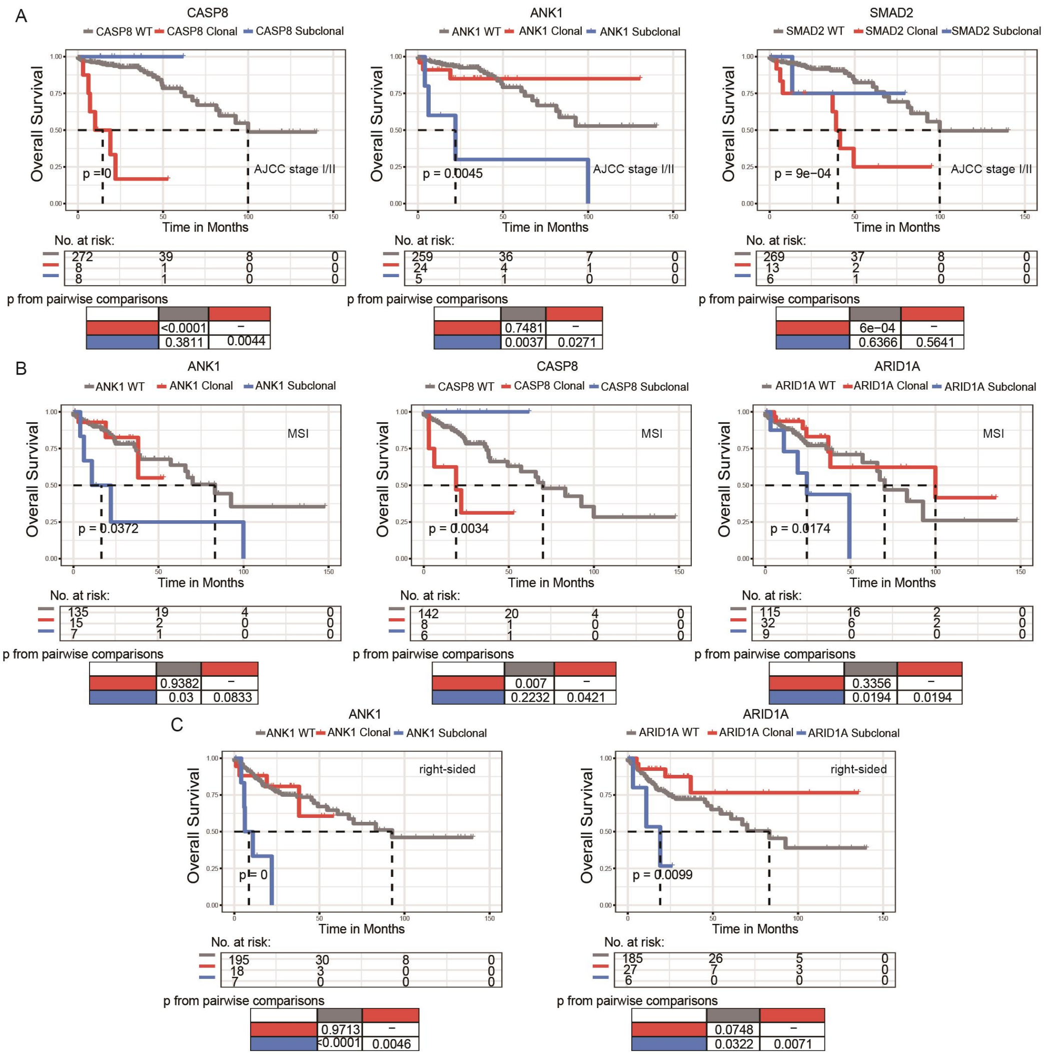

Supplementary Fig. 3. **Subgroup analysis for CRC patients according to the TNM stage, MSI status, and tumor location**. **(a).** Kaplan–Meier estimates overall survival among AJCC stage I/II patients harboring clonal or subclonal *CASP8, ANK1,* and *SMAD2* mutations. (**b).** Kaplan–Meier estimates overall survival in MSI patients harboring clonal or subclonal *ANK1, CASP8, ARID1A* mutations. (**c**). Kaplan–Meier estimates overall survival in right-sided patients harboring clonal or subclonal *ANK1, ARID1A* mutations.

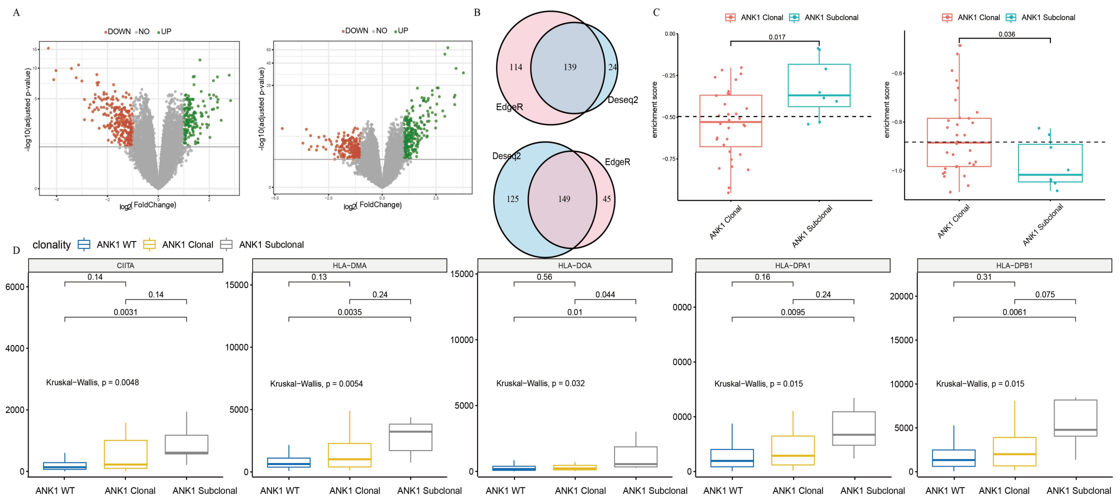

Supplementary Fig. 4. ***ANK1*-mutant gene expression signature**. (a) Differential expression analysis results between patients with ANK1 mutations and without ANK1 mutations are displayed in volcano plots using DESeq2 and EdgeR, where red represents downregulated genes and green represents upregulated genes. (b) Venn diagrams showing the intersection of upregulated genes and downregulated genes, respectively. (c) The difference in ssGSEA enrichment scores of ANK1-mutant upregulated (left panel) and downregulated (right panel) gene expression signature between cases with subclonal ANK1 mutations and cases with ANK1 clonal mutations. P-value is calculated by the Wilcoxon rank-sum test. (d) Differential expression of major histocompatibility complex (MHC) class II and MHC class II transactivator CIITA among CRC patients with clonal ANK1 mutation (yellow), subclonal ANK1 mutation (gray), and ANK1 wide type (blue).

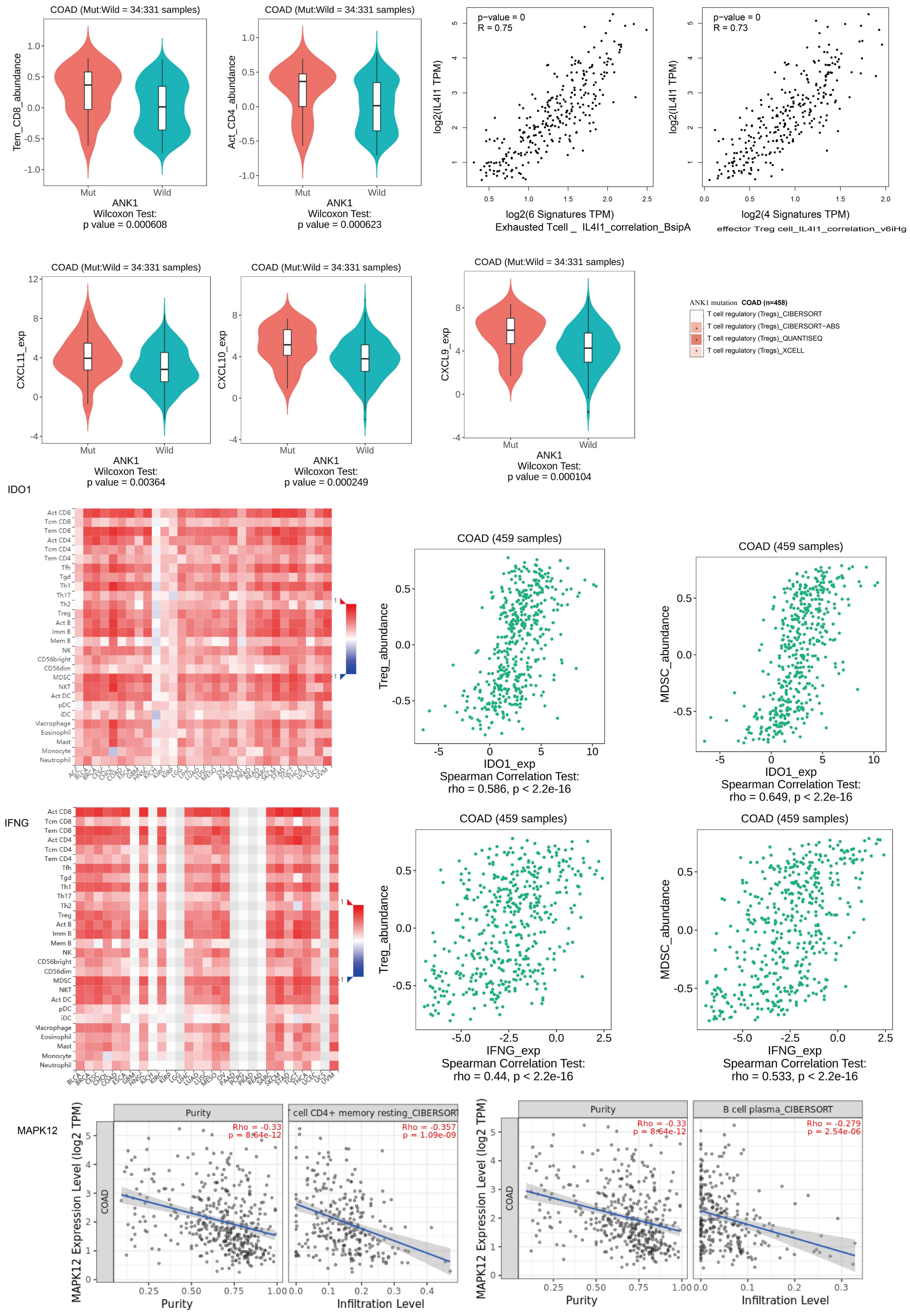

Supplementary Fig. 5. Correlations between of ANK1, ANK1-driven genes IDO1, IFNG, MAPK12 and immune cells in CRC using TISIDB, TIMER and GEPIA.

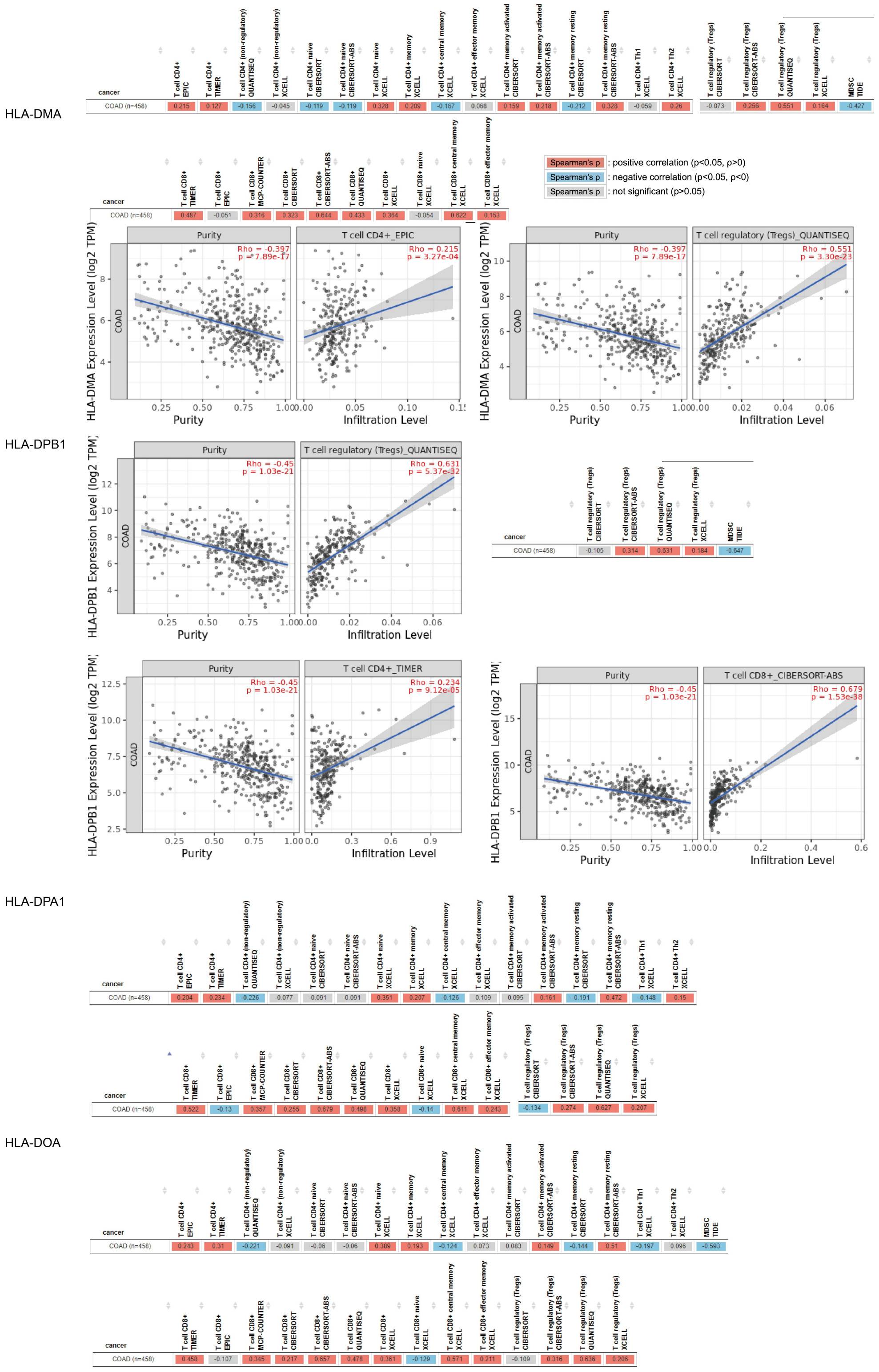

Supplementary Fig. 6. Correlations between of ANK1 subclonal mutation-driven major histocompatibility complex (MHC) class II and immune cells in CRC using TISIDB, TIMER and GEPIA.

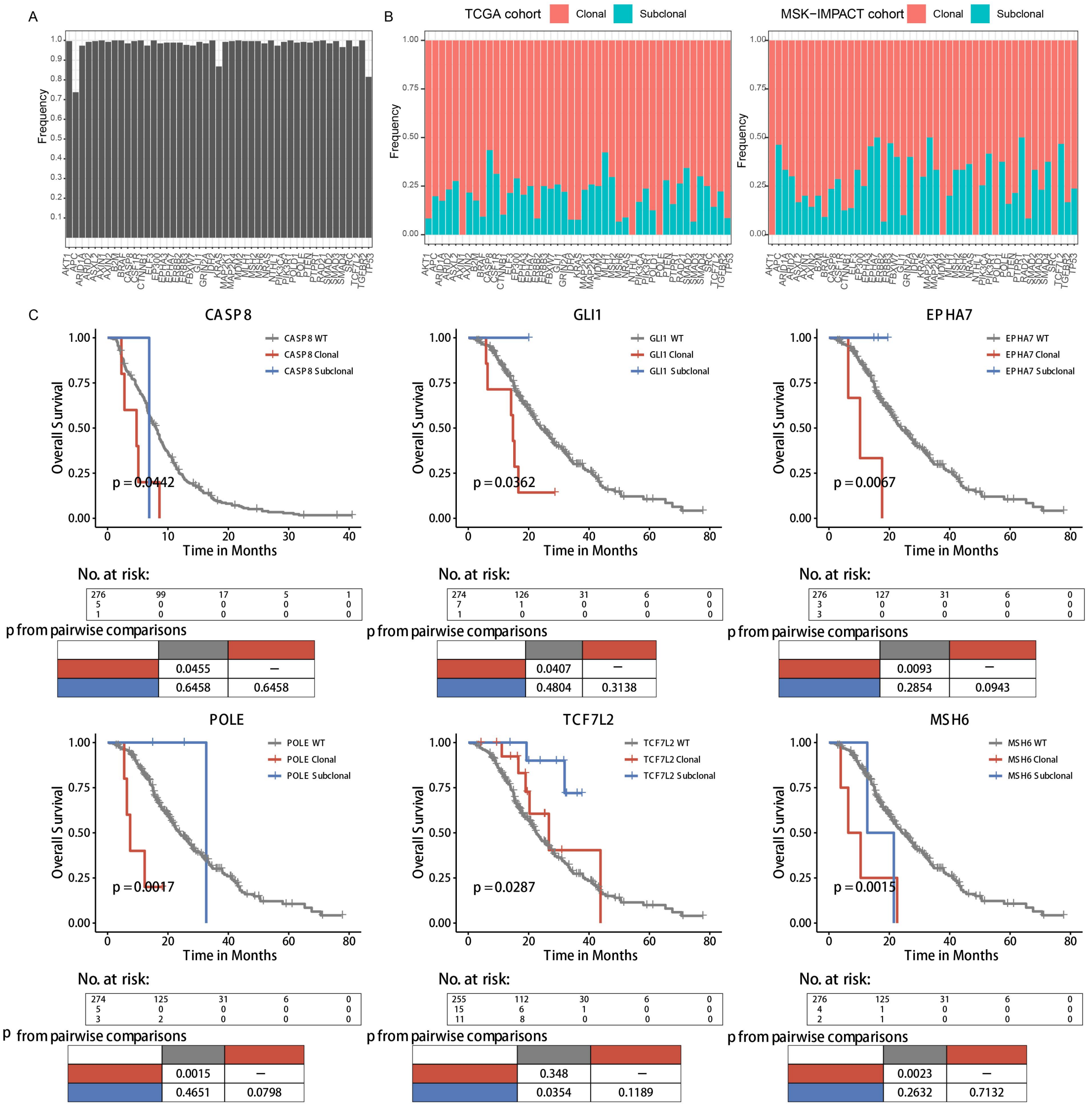

Supplementary Fig. 7 Assessing the consistency of clonality status of driver genes in the validation dataset (A) consistency of clonality of driver genes derived from ABSOLUTE method and approach proposed by McGranahan et al using an independent data set. The y-axis represents the proportion of samples with consistent clonal status of the driver gene. (B) Clonality distribution of mutations in CRC driver genes. The driver genes tend to be clonal in TCGA colorectal cohort (left-panel) and MSK-IMPACT cohort (right-panel). (C) Survival analysis of driver genes in the MSK-IMPACT colorectal cohort. Comparison of overall survival among patients carrying clonal mutations (red line), subclonal mutations (blue line), and patients harboring unmutated genes (gray line) by Kaplan–Meier analysis (with log-rank values) in the cohort of colorectal cancer patients from MSK-IMPACT.

SUPPLEMENTARY TABLES

Supplementary Table 1. Clinical and pathological parameters of patients in TCGA CRC cohort.

Parameters	Patient Num (%)	Parameters	Patient Num (%)
n (%)	536 (100)		
Sex (%)			
Male	279(52.1)	Stage 4	73 (13.6)
Female	257(47.9)	NA	19 (3.5)
MSI status (%)		Age (%)	
MSI	158(29.5)	>= 67.5	268 (50.0)
MSS	376(70.1)	<67.5	268 (50.0)
NA	2(0.4)	Tumour site (%)	
T stage (%)		left-sided	297 (55.4)
T1	17 (3.2)	right-sided	221 (41.2)
T2	97 (18.1)	NA	18 (3.4)
T3	361 (67.4)		
T4	59 (11.0)		
NA	2 (0.4)		
N stage (%)			
N0	308 (57.5)		
N1	133 (24.8)		
N2	93 (17.4)		
NA	2 (0.4)		
M stage (%)			
M0	394 (73.5)		
M1	72 (13.4)		
NA	70 (13.1)		
TNM stage (%)			
Stage 1	95 (17.7)		
Stage 2	196 (36.6)		
Stage 3	153 (28.5)		

Supplementary Table 2. The associations between the clonality of driver genes and the mutation type.

Molecular characteristic	The number of mutations in clonality	The number of mutations in subclonality	P	OR(CI)
APC	Clonal	Subclonal		
Indel	136	66	6.963e-08	0.33 (0.22-0.50)
Others	379	61		
CASP8	Clonal	Subclonal		
Indel	1	8	0.001905	0.033(0.0005-0.43)
Others	10	2		
MLH1	Clonal	Subclonal		
Indel	1	6	0.02128	0.073(0.001-0.82)
Others	13	5		
PTEN	Clonal	Subclonal		
Indel	5	8	0.004845	0.14(0.025-0.66)
Others	29	6		
TP53	Clonal	Subclonal		
Indel	33	8	0.01159	0.299(0.11-0.85)
Others	277	20		
APC	Clonal	Subclonal		
Nonsense	322	42	2.394e-09	3.37(2.20-5.22)
Others	193	85		
PIK3R1	Clonal	Subclonal	0.03311	Inf(1.115381-Inf)
Nonsense	11	0		
Others	15	9		

Supplementary Table 3. Univariate and multivariate analysis of mutation status of driver genes.

	Univariate analysis			Multivariate analysis			Stepwise analysis		
	HR	CI	P	HR	CI	P	HR	CI	P
SMAD2									
MUT vs WT	1.14	0.28-4.63	.02	3.17	1.43-7.04	.0047	3.73	1.84-7.59	<.001
GRIN2A									
Mut vs WT	1.71	1.02-2.86	.04	1.02	0.52-1.98	.96	-	-	-
CASP8									
Mut vs WT	2.26	1.10-4.65	.026	2.09	0.86-5.05	.10	1.98	0.85-4.66	.12
AKT1									
Mut vs WT	2.47	1.20-5.07	.014	1.13	0.42-2.99	.81	-	-	-
AXIN2									
Mut vs WT	1.85	0.99-3.44	.052	1.80	0.76-4.29	.18	-	-	-
BCLAF1									
Mut vs WT	1.95	1.07-3.56	.03	1.91	0.83-4.43	.13	1.98	0.91-4.3	.084
CD70									
Mut vs WT	3.00	1.22-7.78	.02	1.37	0.46-4.11	.57	-	-	-
CNBD1									
Mut vs WT	0.17	0.024-1.23	.08	0.12	0.15-0.94	.04	0.12	0.02-0.92	.04
Age									
>= 67.5 vs < 67.5	2.14	1.44-3.17	2e-04	3.44	2.15-5.51	<.0001	3.51	2.21, 5.58	<.001
Sex									
Male vs Female	1.05	0.73-1.52	.79	0.73	0.47-1.12	.15	0.7	0.46-1.07	.10
Tumour_site									
Left vs Right	0.72	0.49-1.04	.08	0.93	0.58-1.48	.75	-	-	-
M_stage									
M1 vs M0	3.92	2.56-5.99	<.0001	2.67	1.52-4.70	6e-04	2.86	1.70-4.82	<.001
N_stage									
N1/N2 vs N0	2.47	1.70-3.59	<.0001	0.53	0.17-1.68	.28	-	-	-
T_stage									
T3/T4 vs T1/T2	2.46	1.28-4.72	.006	2.17	0.89-5.24	.08	1.96	0.84-4.6	.12
TNM_stage									
StageIII/IV vs Stage I/II	2.70	1.82-4.01	<.0001	5.88	1.70-20.26	.005	3.14	1.81-5.45	<.001
MSI status									
MSI vs MSS	0.80	0.54-1.15	0.22	1.19	0.68-1.85	.66			

Supplementary Table 4. Univariate and multivariate and backward-stepwise cox of the prognostic impact of clinicopathological variables and the clonality of driver genes in pan-cancer.

	Univariate analysis			Multivariate analysis			Stepwise analysis			
	HR	CI	P	HR	CI	P	HR	CI	P	cancer type
NPM1										LAML
Clonal vs WT	2.99	1.302-6.91	0.01	3.40	1.47-7.88	0.004	3.49	1.51-8.06	0.004	
NRAS										LAML
Subclonal vs WT	16.97	2.26-127.49	0.006	33.05	4.29-254.66	<.001	29.11	3.82-222.01	0.001	
Clonal vs WT	1.61	0.22-11.54	0.637	1.16	0.16-8.42	0.883	1.05	0.15-7.59	0.96	
USP44										COAD
Clonal vs WT	9.61	1.30-71.26	0.027	9.38	1.13-77.85	0.038	9.03	1.18-68.9	0.034	
MAP2K1										COAD
Clonal vs WT	23.75	2.90-194.43	0.003	0	0-Inf	0.997	-	-	-	
MAP2K4										COAD
Subclonal vs WT	0	0-Inf	0.996	0	0-Inf	0.997	0	0-Inf	0.997	
Clonal vs WT	13.19	3.08-56.54	<.001	17.06	3.58-81.13	<.001	19.71	4.37-88.88	<.001	
SMAD2										COAD
Subclonal vs WT	4.28	1.03-17.76	0.045	30.778	2.70-350.58	0.006	34.51	3.07-388.13	0.004	
Clonal vs WT	2.01	0.63-6.45	0.240	2.73	0.80-9.24	0.106	2.87	0.86-9.57	0.086	
BAZ1A										SARC
Subclonal vs WT	2.74	0.37-19.86	0.319	2.27	0.31-16.80	0.423	2.24	0.31-16.39)	0.428	
Clonal vs WT	14.065	1.85-106.60	0.011	18.32	2.33-143.72	0.006	18.08	2.34-139.93	0.006	
CDKN2A										SKCM
Subclonal vs WT	3.12	0.99-9.84	0.052	11.60	1.47-91.10	0.020	11.67	1.49-91.19	0.019	
Clonal vs WT	0.62	0.32-1.18	0.143	0.54	0.25-1.19	0.125	0.53	0.24-1.16	0.112	
NFKBIE										SKCM
Clonal vs WT	6.38	0.87-46.67	0.068	11.15	1.41-88.16	0.022	12.27	1.57-95.9	0.017	
PREX2										SKCM

Subclonal vs WT	1.05	0.33-3.31	0.928	0.63	0.08-4.68	0.650	0.57	0.08-4.19	0.58	
Clonal vs WT	0.37	0.21-0.65	<.001	0.35	0.18-0.68	0.002	0.37	0.19-0.71	0.003	
PMS2										READ
Subclonal vs WT	-	-	-	-	-	-	-	-	-	
Clonal vs WT	23.05	1.96-270.07	0.013	0	0-Inf	0.999	-	-	-	
ARID1B										BRCA
Subclonal vs WT	0	0-Inf	0.994	0	0-Inf	0.996	0	0-Inf	0.996	
Clonal vs WT	8.49	1.12-64.23	0.038	10.14	1.30-79.16	0.027	9.72	1.26-74.99	0.029	
BRCA2										BRCA
Subclonal vs WT	19.01	2.51-143.95	0.004	11.48	1.42-92.67	0.022	8.42	1.96-36.06	0.004	
Clonal vs WT	0.90	0.13-6.48	0.918	2.01	0.27-14.79	0.492	0.56	0.08-4.18	0.576	
ERBB2										BRCA
Subclonal vs WT	0	0-Inf	0.996	0	0-Inf	0.999	0	0-Inf	0.999	
Clonal vs WT	3.66	1.33-10.03	0.012	3.88	1.39-10.81	0.009	3.54	1.28-9.79	0.015	
FOXA1										BRCA
Subclonal vs WT	0	0-Inf	0.996	0	0-Inf	0.996	0	0-Inf	0.996	
Clonal vs WT	4.44	1.61-12.24	0.004	5.08	1.80-14.35	0.002	4.87	1.73-13.71	0.003	
RB1										BRCA
Subclonal vs WT	11.27	2.69-47.14	<.001	12.21	2.85-52.34	<.001	12.32	2.87-52.84	0.001	
Clonal vs WT	1.05	0.26-4.27	0.947	1.48	0.35-6.23	0.596	1.46	0.35-6.17	0.604	
BRCA1										BRCA
Subclonal vs WT	6.37	1.57-26.09	0.01	8.94	2.08-38.44	0.003	8.42	1.96-36.06	0.004	
Clonal vs WT	0.47	0.06-3.37	0.453	0.33	0.040-2.71	0.299	0.56	0.08-4.18	0.576	
PMS2										OV
Subclonal vs WT	20.29	2.76-149.31	0.003	51.16	6.26-418.26	<.001	51.16	6.26-418.26	<.001	
Clonal vs WT	0.56	0.08-4.02	0.567	0.61	0.085-4.34	0.618	0.61	0.08-4.34	0.618	

EGFR										LGG
Subclonal vs WT	29.67	6.68-131.86	0	19.31	4.23-88.60	<.001	18.57	4.16-82.89	<.001	
Clonal vs WT	14.25	3.15-64.48	<.001	9.02	1.98-41.01	0.004	8.95	1.97-40.66	0.005	
NF1										LGG
Subclonal vs WT	4.20	1.01-17.54	0.048	2.68	0.63-11.41	0.183	2.49	0.59-10.44	0.213	
Clonal vs WT	4.19	1.65-10.63	0.003	6.66	2.50-17.76	4.189	6.23	2.39-16.24	<.001	
PTEN										LGG
Subclonal vs WT	6.86	2.08-22.65	0.002	7.52	2.24-25.28	0.001	7.47	2.25-24.79	0.001	
Clonal vs WT	4.23	1.51-11.80	0.006	2.31	0.79-6.74	0.125	36.06	4.19-310.06	0.001	
ACVR2A										STAD
Subclonal vs WT	27.89	3.34-232.88	0.002	49.39	5.4-448.23	<.001	44.89	5.1-395.33	0.001	
Clonal vs WT	2.23	0.30-16.28	0.429	1.03	0.13-8.01	0.975	1.05	0.14-8.16	0.961	
CDKN2A										PAAD
Subclonal vs WT	3.98	0.47-33.47	0.203	3.24	0.30-34.81	0.331	2.56	0.26-24.89	0.419	
Clonal vs WT	5.37	1.79-16.13	0.003	12.09	2.74-53.39	0.001	9.2	2.45-34.5	0.001	
CTCF										HNSC
Subclonal vs WT	2.51	1.11-5.71	0.029	2.38	1.02-5.58	0.045	4.09	0.57-29.61	0.163	
Clonal vs WT	4.26	0.59-30.84	0.151	4.21	0.57-31.01	0.159	2.45	1.08-5.57	0.033	
TGFBR2										HNSC
Subclonal vs WT	3.80	1.40-10.31	0.009	3.71	1.32-10.47	0.013	208.66	18.92-2301.17	<.001	
Clonal vs WT	0.97	0.43-2.21	0.954	0.94	0.41-2.14	0.875	-	-	-	
ANK1										KIRC
Subclonal vs WT	1.00E-04	0-Inf	0.997	-	-	-	-	-	-	
Clonal vs WT	8.01	1.12-57.92	0.039	11.48	1.49-87.95	0.019	11.79	1.56-88.93	0.017	
IDH1										GBM
Subclonal vs WT	0	0-Inf	0.988	0	0-Inf	0.988	0	0-Inf	0.988	

Clonal vs WT	0.41	0.21-0.79	0.008	0.47	0.24-0.92	0.027	0.48	0.25-0.93	0.03	
TERT Clonal vs WT	6.63	1.53-28.68	0.011	4.32	0.83-22.44	0.081	5.5	1.15-26.26	0.032	BLCA
